# Long-Term Outcomes of Early Enzyme Replacement Therapy With Asfotase Alfa in Perinatal Benign Hypophosphatasia: Amelioration of Bone Deformities in a Young Child

**DOI:** 10.7759/cureus.78473

**Published:** 2025-02-04

**Authors:** Shuntaro Terayama, Masahisa Kobayashi, Tokumasa Suemitsu, Osamu Samura, Kimihiko Oishi

**Affiliations:** 1 Department of Pediatrics, The Jikei University School of Medicine, Tokyo, JPN; 2 Department of Obstetrics and Gynecology, The Jikei University School of Medicine, Tokyo, JPN

**Keywords:** asfotase alfa, bone deformity, bone spur, hypophosphatasia, short stature

## Abstract

Hypophosphatasia (HPP) is a congenital skeletal dysplasia. Enzyme replacement therapy (ERT) improves survival and bone mineralization of patients with perinatal severe HPP. However, there are few reports of ERT in patients with perinatal benign HPP, and its long-term efficacy remains unclear. Herein, we report the case of a boy with perinatal benign HPP who was initiated on early ERT with asfotase alpha. The patient was suspected of having HPP because of shortened limbs and deformed long bones on fetal 3D-CT. He was diagnosed with HPP based on clinical manifestations, low serum alkaline phosphatase levels, and *ALPL* gene variants. At the age of two years and three months, he had muscle weakness and motor developmental delay requiring support to walk, and ERT was initiated. His muscle strength improved immediately, and he could walk independently two months after starting ERT. Shortening and bowed limbs improved, and his body height increased from -3.48 SD (at the age of two years and three months) to -1.71 SD (at the age of nine years and eight months) after starting ERT. Hence, early ERT effectively improves motor development, bone deformity, and short stature in patients with perinatal benign HPP.

## Introduction

Hypophosphatasia (HPP) is a rare inherited metabolic bone disorder resulting from a deficiency of tissue-nonspecific alkaline phosphatase (ALP), an enzyme essential for bone and tooth mineralization [1,2]. According to the age of onset and clinical severity, HPP is classified into six types: perinatal severe, perinatal benign, infantile, pediatric, adult, and odontohypophosphatasia [2]. The perinatal form of HPP is diagnosed in the neonatal period, with most cases being life-threatening perinatal severe subtype [3]. Enzyme replacement therapy (ERT) with asfotase alfa has been developed to treat HPP and has shown favorable clinical outcomes [4]. Notably, in the most severe form, perinatal severe HPP, the five-year survival rate has significantly improved from 27% in historical controls to 82% in patients receiving treatment [5]. In patients with the pediatric form of HPP, ERT with asfotase alfa has been shown to improve rickets-like bone abnormalities, promote growth, enhance muscle strength, and restore motor function [6]. However, only a limited number of patients with perinatal benign HPP treated with asfotase alfa have been reported, leaving the long-term effects of ERT uncertain [7,8]. Here, we present the long-term clinical outcomes of male patients with perinatal benign HPP who were initiated on ERT with asfotase alpha at the age of two years and three months.

## Case presentation

**Figure 1:**
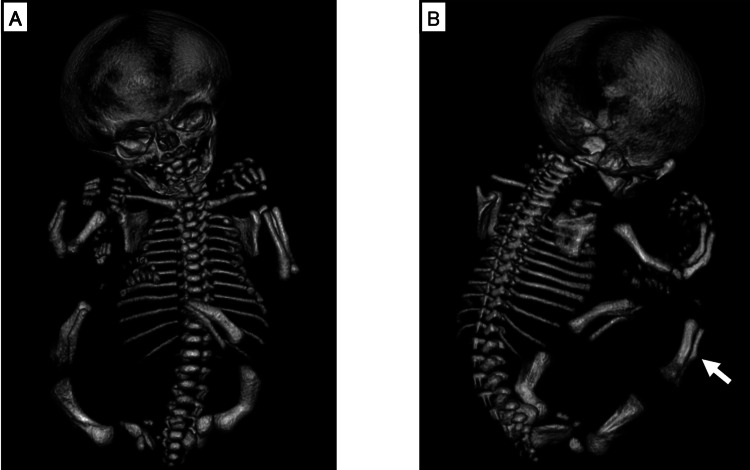
Fetal 3D-CT at 32 weeks gestation A: Deformities of long bones (bowed radius, ulna, femur, and tibia). B: The bone spur of the fibula (arrow). 3D-CT: three-dimensional computed tomography

The patient was a nine-year and eight-month-old boy whose parents had no known consanguinity or family history of genetic disorders. The mother was 38 years old at the time of conception. Fetal ultrasonography at 27 weeks of gestation demonstrated significant bowing and shortening of femurs (-4.7 SD), suggesting congenital skeletal dysplasia. Subsequent fetal 3D-CT at 32 weeks of gestation (Figure 1) revealed long bone deformities and bone spurs of the fibulae, raising suspicion for HPP.

**Figure 2:**
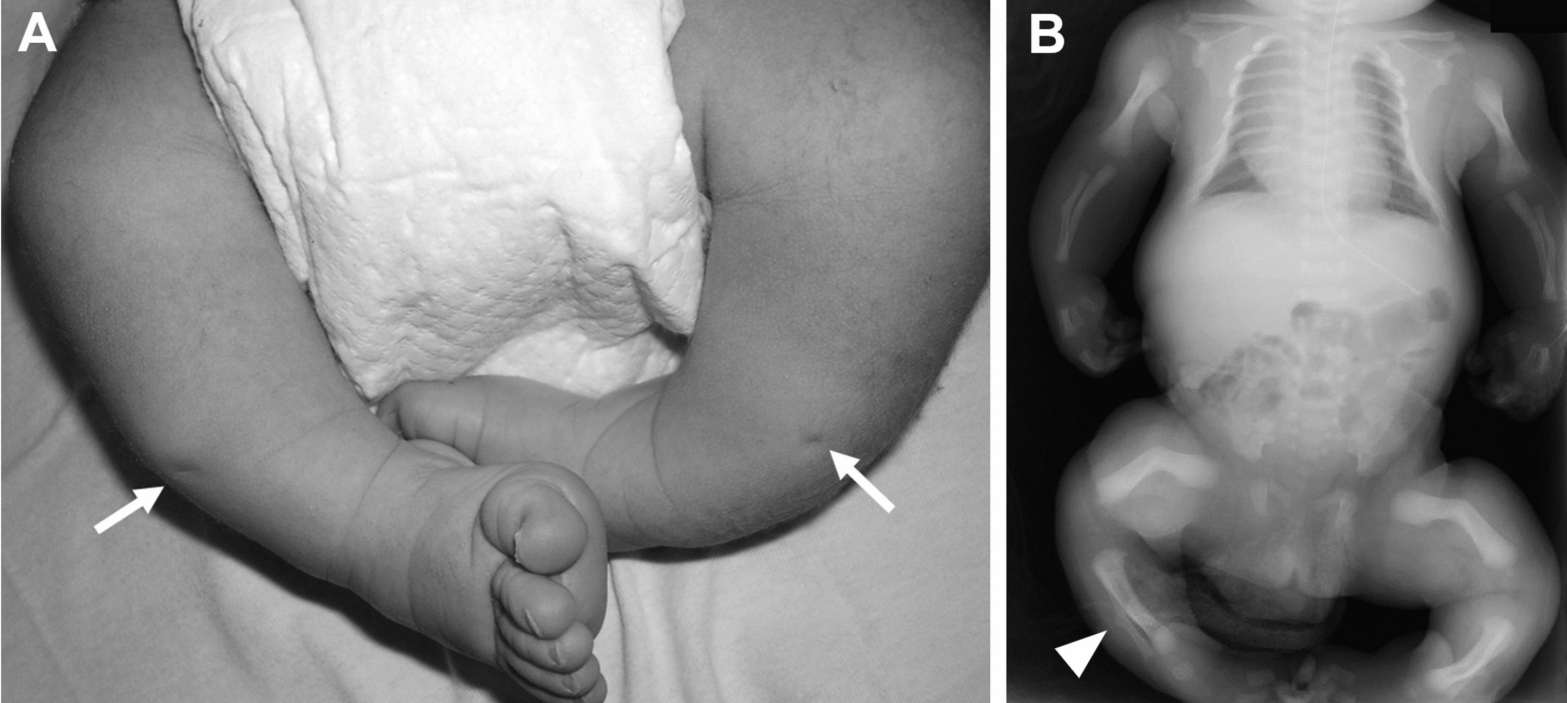
Physical and X-ray findings at birth A. Lower extremities at birth: shortening of lower limbs, the skin dimpling sign (arrow), and bilateral clubfoot. B. X-ray findings at birth: bowed radius, ulna, femur, and tibia. The bone spur of the fibula (arrowhead).

The patient was born by cesarean section at 37 weeks and four days of gestation as the first child of his parents, with a birth weight of 3,599 g and a length of 45.0 cm. At birth, he presented with shortened limbs, flexion contracture of the thighs, skin dimpling of the lower legs, and bilateral clubfoot (Figure 2). Whole-body bone radiographs taken at birth showed deformities of the radius, ulna, femur, and tibia, as well as bone spurs of fibulae. The diagnosis of perinatal benign HPP was confirmed based on a remarkably low serum ALP level of 20 IU/L (normal range for newborn: 190-560) at birth (Table 1) and the identification of compound heterozygous variants with NM_000478.6: c.226C>T (p. Q76*) and NM_000478.6: c.979T>C (p.F327L) in the *ALPL* genes.

**Table 1:** Laboratory results at birth The serum ALP level was significantly decreased. *normal range for newborns. WBC: white blood cell count, RBC: red blood cell count, Hb: hemoglobin, Ht: hematocrit, Plt: platelet count, pH: potential of hydrogen (measure of acidity/alkalinity), pCO₂: partial pressure of carbon dioxide, pO₂: partial pressure of oxygen, HCO₃⁻: bicarbonate, BE: base excess, Glu: glucose, AST: aspartate aminotransferase, ALT: alanine aminotransferase, LDH: lactate dehydrogenase, ALP: alkaline phosphatase, TP: total protein, Alb: albumin, CK: creatine kinase, BUN: blood urea nitrogen, Cr: creatinine, Na: sodium, K: potassium, Ca: calcium, IP: inorganic phosphate phosphorus

Complete blood count	Patient values	Normal range
WBC	13100 /μL	(6000-18000*)
RBC	435×10^6^ /μL	(288-651×10^6^*)
Hb	15.4 g/dL	(13.2-22.0*)
Ht	44.40%	(39.5-66.5*)
Plt	190×10^3^ /μL	(100-260×10^3^*)
Blood gas analysis (arterial)	Patient values	Normal range
pH	7.354	-
pCO2	36.1 mmHg	-
pO2	86 mmHg	-
HCO3-	19.7 mmol/L	-
BE	-5.1	-
Glu	58 mg/dL	-
Biochemical analysis	Patient values	Normal range
AST	21 IU/L	(13-30)
ALT	8 IU/L	(10-42)
LDH	276 IU/L	(124-222)
ALP	20 IU/L	(190-560*)
TP	4.7 g/dL	(4.4-6.3*)
Alb	3.3 g/dL	(3.3-4.5*)
CK	226 IU/L	(39-248)
BUN	8 mg/dL	(8-20)
Cr	0.46 mg/dL	(0.3-0.9*)
Na	137 mmol/L	(128-145)
K	4.2 mmol/L	(3.6-4.8)
Ca	9.4 mg/dL	(8.8-10.1)
IP	5.7 mg/dL	(4.5-8.8)

**Figure 3:**
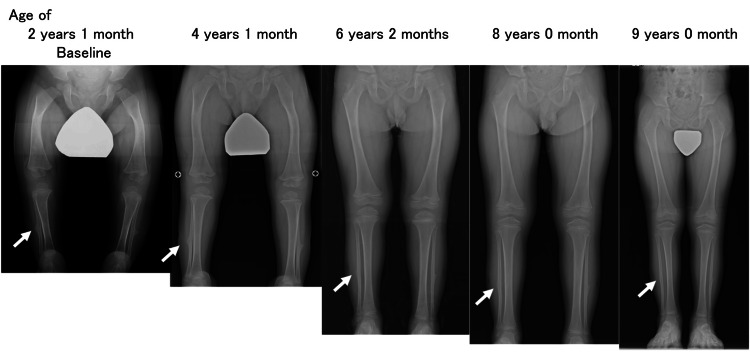
Changes in lower extremity radiographic findings after initiating asfotase alfa Improvement in femoral deformity, bow legs, and bone spur of the fibula (arrow).

At the age of four months, he underwent corrective surgery for bilateral clubfoot. He exhibited motor developmental delays due to limb deformities and muscle weakness, achieving milestones such as rolling over at the age of nine months, sitting independently at 14 months, pulling up to stand at 18 months, and walking with support at 21 months. At the age of two years and three months, following the approval of asfotase alfa for HPP in Japan in 2015, ERT with asfotase alfa was initiated. He showed a rapid improvement in muscle strength shortly after starting ERT and could stand without support after one month and walk independently after two months. No episodes of hypocalcemia or seizures were observed at the initiation of asfotase alfa therapy. Six years and nine months after starting the ERT (at the age of nine years), the femoral curvature and bone spurs of the fibulae had completely resolved (Figure 3).

**Figure 4:**
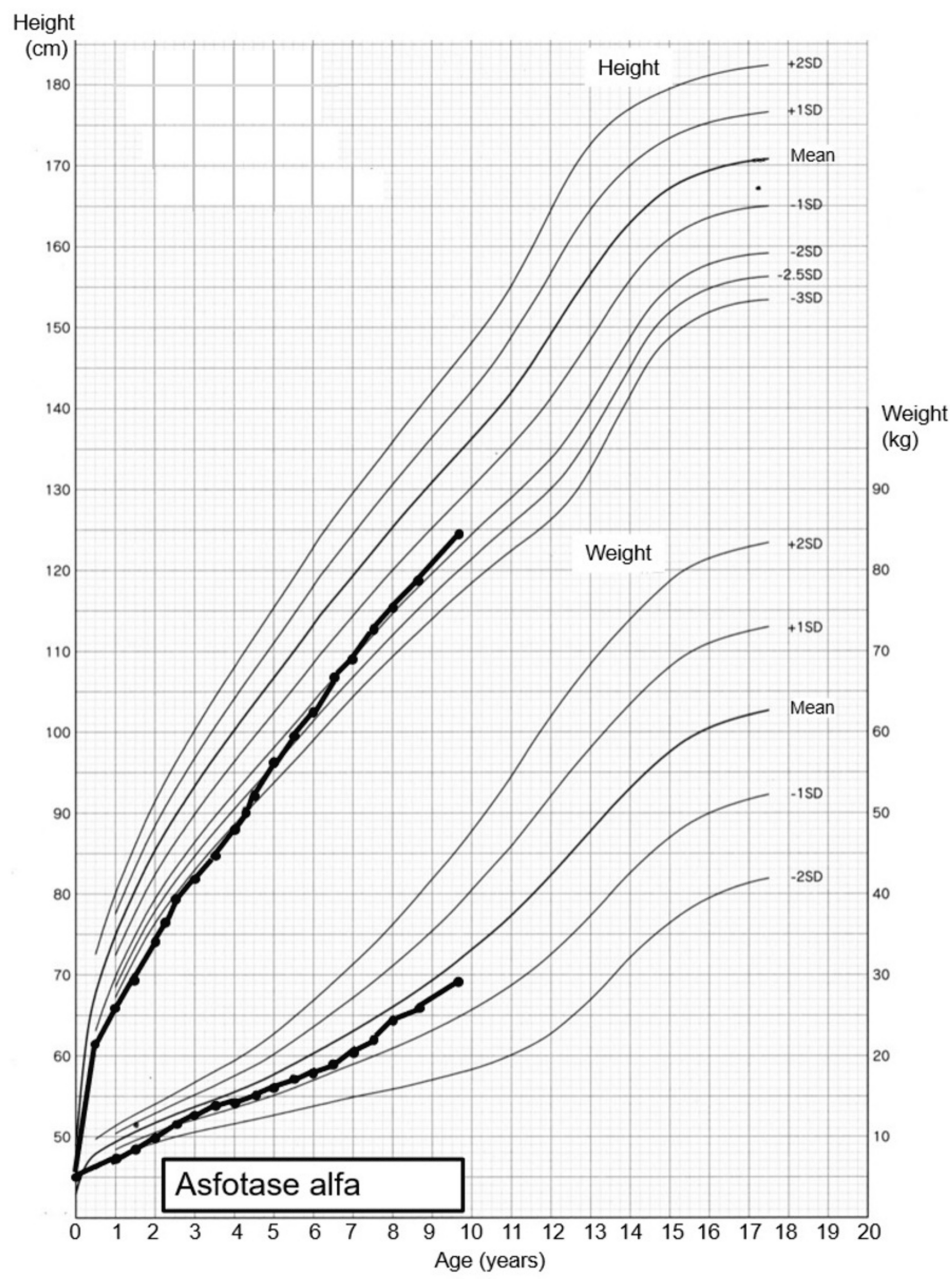
Patient growth chart Body height increased from -3.48 SD at baseline to -1.71 SD at seven years and six months after starting ERT. SD: standard deviation, ERT: enzyme replacement therapy

His height improved significantly from -3.48 SD (76.8 cm at two years and three months) at baseline to -1.71 SD (124.6 cm at nine years and eight months) at seven years and six months after starting ERT (Figure 4). No adverse events, including hypocalcemia, were observed.

## Discussion

According to a Japanese report characterizing the frequencies of each form of HPP among nearly 100 cases, the perinatal severe and benign forms are the most common in Japan, accounting for 45.9% and 22.4% of cases, respectively [3]. Okazaki et al. described the case of a newborn girl with severe perinatal HPP who was diagnosed prenatally and initiated on ERT on the first day of life [9]. Despite presenting with severe bone hypomineralization and respiratory failure at birth, the patient demonstrated significant improvement in bone mineralization within three months, was weaned from mechanical ventilation, and survived for over one year.

Although outcomes for more severe forms of HPP, such as perinatal severe, have been reported, information on the ERT outcomes of the perinatal benign form treated with asfotase alfa remains limited despite its relatively high prevalence within the disorder spectrum. Notably, the one year of treatment with asfotase alfa shortly after birth showed improvements in bowing deformities [7]. These findings highlight the potential importance of early intervention with ERT in addressing bone pathology. Conversely, one male patient with the perinatal benign form was treated with asfotase alfa for approximately three years from the age of seven years, representing the only reported case with a relatively long follow-up period [8]. This case showed limited effects, with no significant improvements in bone mineral density or limb bowing deformities. In contrast, our case represents the most extended follow-up period reported to date, with nearly eight years of ERT with asfotase alfa. The treatment led to a remarkable resolution of femoral deformities and catch-up growth, with the patient achieving a height appropriate for his age and significant improvements in motor development. Based on these observations, along with the improvements observed during the long-term follow-up of our case, several key insights regarding ERT with asfotase alfa for this form of HPP can be highlighted: (1) ERT can be effective for the perinatal benign form of HPP. (2) Even when ERT is not initiated at birth, starting treatment as soon as diagnosis appears beneficial with a significant potential for improving bone phenotypes. (3) Long-term use of ERT may contribute to enhanced physical growth.

Hypocalcemia, which can trigger seizures, has been previously reported in patients with the perinatal severe form of HPP receiving ERT [4]. In contrast, the perinatal benign form has not been associated with severe hypocalcemia during ERT, possibly due to differences in the extent of “the hungry bone effect” following treatment. In our patient, despite long-term ERT with asfotase alfa, serum calcium and phosphorus levels remained within normal limits, suggesting that the risk of these electrolyte abnormalities may be minimal in perinatal benign HPP. However, careful monitoring is still warranted.

Asfotase alfa is a newly developed recombinant enzyme, and its frequent injections over an extended period can impose high costs at this time, depending on insurance coverage in each county. This financial burden may create significant social and personal challenges. However, the treatment has the potential to alleviate psychological distress by enabling better participation in physical activities, resulting in overall well-being in affected children and potentially reducing long-term medical costs associated with complications of HPP.

With the advent of high-resolution fetal imaging technologies, detecting congenital malformations before birth has become increasingly feasible. Fetal ultrasound is a valuable first-line tool, with congenital skeletal dysplasia strongly suspected when femur length measures below -3.0 SD [10,11]. However, diagnosing the perinatal benign form remains challenging due to its milder phenotype and subtle findings. Abnormalities on ultrasonography are often less pronounced, with bone hypomineralization rarely observed. Instead, the primary indicators may be morphological abnormalities such as limb shortening and deformity [7]. Recently, 3D-CT has also become available for the prenatal diagnosis of congenital skeletal dysplasia, including HPP [12-14]. This advanced imaging modality can significantly aid in more accurate diagnosis and management planning, helping to reduce complications associated with skeletal dysplasia. For instance, babies with skeletal dysplasia often face risks of respiratory failure and bone fracture, making the early detection of bone malformations critical for comprehensive care planning. As demonstrated in our case, HPP could be identified prenatally from fetal 3D-CT findings. Since delayed initiation of ERT can lead to progressive bone deformities, restricted height potential due to growth plate closure with age, and impaired physical function, early intervention with an accurate diagnosis is highly likely to provide optimal benefits by promoting proper skeletal development, enhancing mobility, and improving overall functional outcomes. These observations highlight the importance of integrating fetal 3D-CT imaging when abnormal bone findings are detected on ultrasonography. Such an approach can facilitate timely diagnosis and preparation for the rapid initiation of available therapies, potentially improving outcomes for affected infants.

One of the variants identified in this case, c.979T>C (p.F327L) in the *ALPL* gene, is a common allele in Japan associated with a milder phenotype and frequently observed in perinatal benign HPP [3,15]. The clinical phenotype in such cases often depends on the pathogenicity of the second allele. In our case, the second allele, c.226C>T (p.Q76*), is a previously unreported nonsense variant predicted to result in a complete loss of enzymatic activity and is considered to be associated with a severe phenotype. The combinations of compound heterozygous variants with p.F327L and loss of function variant can give rise to a wide range of phenotypes other than perinatal severe form [15], highlighting the complexity of genotype-phenotype correlations in HPP. According to the Japanese Multi-Omics Reference Panel (jMorp, https://jmorp.megabank.tohoku.ac.jp/), the allele frequency of the p.F327L variant is 0.002703, which is relatively high in the general Japanese population. This suggests that HPP may occur more frequently than previously anticipated, particularly in milder phenotypes. Consequently, clinicians should maintain a high index of suspicion for this condition when abnormal bone findings are observed.

## Conclusions

Early intervention with ERT effectively improves growth disturbances and bone deformities, even in perinatal benign HPP. However, due to the limited number of reported early treatment cases, further case studies are needed to understand its long-term outcomes better. Moreover, prenatal diagnosis is important to maximize the effectiveness of ERT in perinatal benign HPP. Fetal 3D-CT imaging could make prenatal diagnosis more accurate in congenital skeletal dysplasia.
